# Evaluation of Reliability of Formulas for Intraocular Lens Power Calculation After Hyperopic Refractive Surgery

**DOI:** 10.3390/jcm14061990

**Published:** 2025-03-15

**Authors:** Rosa Boccia, Michele Lanza, Giuseppe Luciano, Italo Fattore, Luigi Serra, Salvatore Ambrosio, Francesco Abbate, Francesca Simonelli

**Affiliations:** Multidisciplinary Department of Medical, Surgical and Dental Specialities, Università della Campania Luigi Vanvitelli, 80132 Naples, Italy; rosa_boccia111@hotmail.com (R.B.); lucianogiuseppe@hotmail.it (G.L.); doc.italofattore@gmail.com (I.F.); luigi.serra5@gmail.com (L.S.); ambrosio.salvatore2@gmail.com (S.A.); abbatefrancesco.92@gmail.com (F.A.); francesca.simonelli@unicampania.it (F.S.)

**Keywords:** hyperopic refractive surgery, PRK, LASIK, IOL power calculation, IOL formulas

## Abstract

**Background:** We evaluate the accuracy of intraocular lens (IOL) power calculation in the following formulas—Barrett True-K No History (BTKNH), EVO 2.0 Post-Hyperopic LASIK/PRK (EVO 2.0), Haigis-L, Pearl-DGS, and Shammas (SF)—with patients who have undergone cataract surgery at the Eye Unit of University of Campania Luigi Vanvitelli, Naples, Italy, and had prior hyperopic laser refractive surgery. **Methods:** A monocentric, retrospective, comparative study, including the charts of patients who had undergone cataract surgery and previous hyperopic laser refractive surgery, was retrospectively reviewed. Patients with no other ocular or systemic disease which might interfere with visual acuity results and no operative complications or combined surgery were enrolled. The mean absolute prediction error (MAE) was calculated for each formula and compared. Subgroup analysis based on the axial length and mean keratometry was performed. **Results:** A total of 107 patients (107 eyes) were included. The MAE calculated with SF provided less accurate (*p* < 0.05) results when compared to both BTKNH and EVO 2.0 formulas. The MAE obtained using Haigis-L, EVO 2.0, Pearl-DGS, and BTKNH showed no significant differences. **Conclusions:** The analysis of the accuracy of the selected formulas shows no clear advantage in using one specific formula in standard cases, but in eyes where it is mandatory to reach the target refraction, SF should be avoided.

## 1. Introduction

Patients undergoing cataract surgery today have higher expectations and may be disappointed if their post-operative refraction deviates from their goals [1,2]. As a result, physicians’ preoperative evaluations have become highly detailed in order to achieve a calculation of the intraocular lens (IOL) to be implanted which is as accurate as possible [1,2]. Many sources of error have been detected and strategies to overcome them have been proposed [1,2]. One of the ocular features that has proved most difficult when attempting to reach target refraction before cataract is prior laser vision correction (LVC) [3,4,5]. The difficulty lies in the calculation of accurate corneal refractive power because of the altered anterior to posterior corneal power ratio, and unreliable prediction of the effective lens position (ELP) due to the inclusion of the inaccurate corneal curvature [4,5]. Methods and formulas have been proposed to improve the accuracy of IOL calculation in these eyes and many studies have been published focusing mainly on the eyes, which have undergone prior myopic refractive surgery [6,7,8,9,10,11,12,13,14,15,16,17,18,19,20]. Although many formulas have been proposed to improve IOL calculation accuracy, in eyes that have undergone hyperopic refractive surgery as well, there is a lack of studies focusing on the comparison of the results obtained using these formulas [21,22,23].

Although hyperopic refractive surgery is less common than myopic surgery, due to developments in the technology dedicated to both the diagnosis and treatment of this kind of refractive defect, the number of surgeries is likely to increase; thus, many more patients are expected to have these characteristics in the near-future. For this reason, studies comparing the accuracy of formulas in calculating the IOL power after hyperopic LVC are needed because there is a lack of information about this topic compared to current knowledge about the problem of IOL power calculation after myopic LVC.

In eyes that had undergone hyperopic refractive surgery, there was an overestimation of the IOL to be implanted during cataract surgery (when using formulas developed for standard eyes), leading to a post-operative myopic refraction that was, generally, better tolerated in comparison to hyperopic defects. When a patient demands emmetropia or in the case of a premium IOL implant, this kind of error is less easily tolerated [1].

The purpose of this study is to compare, through a large cohort of eyes which have undergone implantation of a single type of IOL by the same surgeon, the accuracy of some of the most frequently used formulas for the calculation of IOL power in eyes which have undergone hyperopic refractive surgery with an excimer laser: Barrett True-K No History (BTKNH), EVO 2.0 Post-Hyperopic LASIK/PRK (EVO 2.0), Haigis-L, Pearl-DGS, and Shammas (SF). This would be one of the first studies evaluating a large cohort of patients with this many formulas; moreover, this study is among the first to provide subgroup analysis according to axial length (AL) and mean keratometry (K) values.

## 2. Materials and Methods

In this retrospective study, 107 charts of patients who underwent cataract surgery at the Eye Unit of the University of Campania Luigi Vanvitelli and who had previously undergone hyperopic laser refractive surgery (either PRK or LASIK) were reviewed. The Institutional Review Board of the University of Campania “Luigi Vanvitelli” (Azienda Ospedaliera Universitaria, Università degli Studi della Campania, “Luigi Vanvitelli”, 0012575/2020) approved this study and each patient provided informed consent both for the surgery and for the use of their data for further research. This study adheres to the tenets of the Declaration of Helsinki.

All patients had a complete eye visit which included K, anterior chamber depth, and AL measurements performed with the IOL Master 700 (Carl Zeiss Meditec AG, Jena, Germany, software version 4.08.002).

All surgeries were performed under topical anesthesia by the same surgeon (M.L.) using a temporal clear corneal incision (2.4 mm) and phacoemulsification technique with in-the-bag IOL placement.

The included patients had a cataract which significantly impaired visual acuity, had undergone prior LVC for a hyperopic defects, and presented no other ocular or systemic disease which might impede visual acuity recovery. Exclusion criteria were intraoperative or post-operative complications, optimum post-cataract surgery with corrected distance visual acuity < 20/40, ultrasound biometry having been utilized to measure AL, combined surgery such as phaco-trabeculectomy for glaucoma, or phaco-vitrectomy for epiretinal membrane or macular holes. The SN60AT IOL (Alcon Laboratories Inc., Forth Worth, TX, USA) was implanted in each patient, using the constant suggested by the User Group for Laser Interference Biometry (ULIB), and the Haigis-L formula was selected with the objective of emmetropia in every eye. The implant of this IOL would provide an overall improvement in the quality of the vision of the patients because of the compensation of some of the negative aberration observed after hyperopic LVC with a positive aberration design.

The refraction collected at the 8-week follow-up after uneventful cataract surgery was used for statistical analysis. In addition, calculations using the observed refractive results were performed with BTKNH, EVO 2.0, Haigis-L, Pearl-DGS, and SF. The EVO 2.0 and Pearls-DGS results were calculated using formula-specific websites, whereas the BTKNH, Haigis-L, and SF results were obtained through IOL Master [24,25,26,27].

The prediction error for each formula was calculated as actual post-operative refraction (calculated as spherical equivalent) minus the predicted refraction for each formula for the implanted IOL power. The mean numerical prediction error (ME), mean absolute prediction error (MAE), standard deviation of the prediction error (SDME), and median absolute prediction error (MedAE) were calculated for each formula as well as the percentage of eyes that had a prediction error of ±0.50 and ±1.00 D.

Further subgroup analysis was performed in relation to subgroups of AL and K. Group 1 included eyes with an AL ≤ 21.0 mm, Group 2 with an AL between 21.01 mm and 22.0 mm, and Group 3 with an AL > 22.0 mm. Group A included eyes with a K ≤ 46.0 D, Group B with a K ranging from 46.01 D to 49.0 D, and Group C including eyes with a K of >49.0 D. The division of the subgroups was aimed at obtaining statistically comparable cohorts.

### Statistical Analysis

As recommended by Wang et al. [25] in their JCRS editorial, statistical analyses were performed using the Friedman test to assess for differences in absolute error between formulas and, in the event of a significant result, post hoc analysis was undertaken using the Wilcoxon signed-rank test with Bonferroni correction. The McNemar test with Bonferroni correction was used to assess for statistical significance between the percentage of eyes within a prediction error of ±0.50 D and ±1.00 D. All statistical analyses were performed using SPSS software (version 21.0, IBM Corp, Armonk, NY, USA.).

## 3. Results

1. A total of 107 eyes of 107 patients were included in this study. If patients underwent cataract surgery in both eyes, only the first eye was included in the study to avoid bias in the calculation of the IOL of the second eye in relation to knowledge of the refractive result of the first.
2. The eyes included in the study had a mean AL of 21.64 ± 0.91 mm, a mean KM of 47.78 ± 2.07 D, and a mean ACD of 3.1 ± 0.36 mm.
3. The overall analysis showed that every formula provided a low level of myopic refraction in the evaluated cohort; the MAE calculated with SF provided less accurate (*p* < 0.05) results compared to both BTKNH and EVO 2.0 formulas; the MAE obtained using Haigis-L, EVO 2.0, Pearl-DGS, and BTKNH showed no significant differences; and all the formulas that were evaluated reported no significant differences in obtaining refraction in the ±0.50 D and ±1.00 D (*p* > 0.05) ranges (Table 1).

4. With regard to AL and SL, the accuracy of the formulas in the group of eyes shorter than 21 mm demonstrated a lower accuracy level when compared with BTKNH and Haigis-L; EVO 2.0 showed a higher (*p* < 0.05) MAE compared to BTKNH; Pearl-DGS showed no significant MAE differences with the other formulas tested; and all the formulas evaluated reported no significant differences in obtaining refraction within the ±0.50 D and ±1.00 D (*p* > 0.05) ranges (Table 2).

5. In the group of eyes with AL ranging from 21 to 22 mm, Pearl-DGS showed a higher MAE (*p* < 0.05) compared to EVO 2.0; no other significant differences were observed when comparing the results obtained with the tested formulas; and all the formulas evaluated reported no significant differences in obtaining refraction in the ±0.50 D and ±1.00 D (*p* > 0.05) ranges (Table 2).
6. In the eyes with AL greater than 22 mm, SF showed a higher (*p* < 0.05) MAE compared to Pearl-DGS, whereas no other significant differences were detected in the other formulas, and Haigis-L and EVO 2.0 formulas showed the highest percentage of eyes with refraction in the ±1.00 D (*p* < 0.05) range (Table 2).
7. In the evaluation of the accuracy of formulas according to anterior corneal power, in the eyes with K < 46 D, the evaluated formulas did not show differences in MAE, whilst no significant differences were observed in the percentages of eyes obtaining refraction within the ±0.50 D and ±1.00 D (*p* > 0.05) ranges (Table 3).

8. In the eyes with a K ranging from 46 to 49 D, SF showed a higher (*p* < 0.05) MAE compared to BTKNH; no other significant differences were detected in the MAE provided by the other formulas; and all the formulas evaluated reported no significant differences in obtaining refraction within the ±0.50 D and ±1.00 D (*p* > 0.05) ranges (Table 3).
9. In eyes with a K higher than 49 D, SF provided a higher (*p* < 0.05) MAE in comparison to both BTKNH and EVO 2.0 formulas; no other significant difference was detected when comparing the MAE of the tested formulas; and all the formulas evaluated reported no significant differences in obtaining refraction within the ±0.50 D and ±1.00 D (*p* > 0.05) ranges (Table 3).

## 4. Discussion

In recent years, we have seen many innovations, leading to the possibility of providing our patients with very good spectacle-free vision after cataract surgery, thanks to improvements in diagnostics and formulas to calculate IOL and materials of artificial lenses, changing the expectations of both patients and physicians [1,2]. With advancements in IOL technology, numerous models are now available, offering excellent vision for both distance and near viewing [26].

To maximize the properties of these IOLs and to avoid patient disappointment, accurate IOL power calculation is mandatory [2,26]. Because some eyes have specific characteristics that impede emmetropia or the desired target refraction, this may not always be achieved [1]. This is the case particularly in eyes that have previously undergone hyperopic laser refractive surgery, a type of surgery that was rare in the past, but it is important to remember that it is becoming ever more frequent, and it is important to highlight that they are always growing in number too [3,4,5].

Many methods have been proposed in an attempt to avoid these difficulties, and many studies have been published, although tending to focus more on myopic rather than hyperopic surgery [6,7,8,9,10,11,12,13,14,15,16,17,18,19,20]. Very few studies compare the results of different IOL power calculation formulas in previously hyperopic eyes [21,22,23].

One of the common characteristics in these patients is that they undergo cataract surgery many years after LVC was carried out, so it is difficult for physicians to know what their condition was prior to the first surgery. Consequentially, in this study, only formulas not requiring pre-hyperopic LVC data were selected.

The results observed in this study show that in the overall population analyzed, SF showed a lower MAE even though no significant difference was observed in providing a refraction in the ±0.50 D and ±1.00 D (*p* > 0.05) ranges in the formulas evaluated.

Similar behaviors were also observed in the different subgroups evaluated with some minor differences: Haigis-L and EVO 2.0 formulas provided higher percentages in eyes longer than 22 mm with refraction within the ±1.00 D (*p* < 0.05) range.

When comparing these data with those of previously published studies, it can be noted that Francone et al. did not highlight one formula as being more accurate than the others under evaluation, although Holladay 2 provided more eyes with a refraction of ±0.50 D, in a multicenter study including 39 eyes of 24 patients, thus introducing more than one bias in the data analysis [22].

Vrijman et al. evaluated the ASCRS online calculator including both the formulas requiring pre-LVC data and those that did not [21]. Upon evaluating 64 eyes of 38 patients, they did not observe one formula that demonstrated greater accuracy than the others; however, the Masket Formula showed a higher (*p* < 0.05) MAE [11].

Hamil et al. confirmed the lack of superiority of the formulas tested, both those that required pre-LVC data and those that did not, in 21 eyes of 21 patients [23].

This study has some limitations such as its retrospective nature and its relatively small cohort. For this reason, the authors strictly adopted more accurate strategies to analyze and compare data in this topic, aiming to provide results that were as reliable as possible [25]. Aiming to improve the overall quality of the study, only the first eye that underwent cataract surgery after hyperopic LVC was included, avoiding bias related to the knowledge of the refractive result when the second eye was treated. A comparative, prospective study would provide interesting results and would be considered to unveil the more complicated aspects of this topic.

The improvements in the overall quality of the LVC observed in recent years are pushing both patients and physicians to select more accurate solutions, aiming to provide the best vision for everyone. To perform these better selections, it is important to have data supporting them, and this study could be useful because there is a lack of information about the formulas to calculate IOL power after hyperopic LVC. The results observed help physicians in better selecting the proper formula in the selected eyes.

In conclusion, this study evaluates the largest cohort of patients that have undergone cataract surgery following hyperopic LVC. The overall accuracy analysis performed in this study shows no clear advantage to using most of the tested formulas, but aiming to reach the best refractive results, SF should be avoided, and Haigis-L and EVO 2.0 formulas should be adopted for eyes with an AL greater than 22 mm.

These data need to be confirmed through further, larger-population studies, which would allow subgroup analysis to be performed, thus providing more accurate indications, which would be invaluable to physicians in more complex cases.

## Figures and Tables

**Table 1:** Overall outcomes for each formula included in this study. Bold values had statistical difference.

Formula	MAE ^3^	ME ^4^	SD ^5^	MedAE ^6^	±0.50 D (%)	±1.00 D (%)
BTKNH ^1^	0.412	−0.132	0.553	0.255	65.4	87.9
SHAMMAS	**0.460**	−0.127	0.600	0.300	63.6	86.9
Haigis-L	0.435	−0.127	0.596	0.280	67.3	89.7
EVO 2.0 ^2^	0.422	−0.142	0.563	0.260	66.4	89.7
Pearl-DGS	0.435	−0.143	0.572	0.273	64.5	88.8

^1^ Barrett True-K no history; ^2^ EVO 2.0 Post-Hyperopic LASIK/PRK; ^3^ mean absolute prediction error; ^4^ prediction error; ^5^ standard deviation; ^6^ median absolute prediction error.

**Table 2:** Outcomes for each formula according to axial length subgroup. Bold values show statistical significance.

Group	BTKNH ^3^	Shammas	Haigis-L	EVO 2.0 ^4^	Pearl-DGS
**≤21 mm (*n* = 30)**					
±0.50 D (%)	70.00	66.70	70.00	70.00	70.00
±1.00 D (%)	73.30	73.30	76.70	73.30	76.70
MAE ^1^	0.480	**0.554**	0.493	0.524	0.505
MedAE ^2^	0.245	0.340	0.252	0.240	0.294
**21–22 mm (*n* = 34)**					
±0.50 D (%)	79.40	82.35	76.47	79.40	76.47
±1.00 D (%)	88.20	88.20	91.20	91.20	85.30
MAE ^1^	0.308	0.316	0.332	0.286	**0.335**
MedAE ^2^	0.140	0.150	0.137	0.130	0.137
**>22 mm (*n* = 43)**					
±0.50 D (%)	51.20	48.80	55.80	53.50	48.80
±1.00 D (%)	97.70	95.30	97.70	100.00	100.00
MAE ^1^	0.447	0.509	0.476	0.458	0.463
MedAE ^2^	0.390	0.510	0.465	0.400	0.509

^1^ Mean absolute prediction error; ^2^ median absolute prediction error; ^3^ Barrett True-K no history; ^4^ EVO 2.0 Post-Hyperopic LASIK/PRK.

**Table 3:** Outcomes for each formula according to mean keratometry subgroup. Bold values show statistical significance.

Group	BTKNH ^3^	Shammas	Haigis-L	EVO 2.0 ^4^	Pearl-DGS
**≤46 mm (*n* = 22)**					
±0.50 D (%)	59.10	54.50	63.60	59.10	54.50
±1.00 D (%)	86.40	90.90	90.90	90.90	86.40
MAE ^1^	0.468	0.484	0.461	0.468	0.512
MedAE ^2^	0.305	0.340	0.315	0.275	0.387
**46–49 D (*n* = 58)**					
±0.50 D (%)	67.20	65.50	69.00	69.00	67.20
±1.00 D (%)	94.80	93.10	94.80	96.60	96.60
MAE ^1^	0.367	0.417	**0.399**	0.372	0.377
MedAE ^2^	0.230	0.285	0.280	0.262	0.199
**>49 D (*n* = 27)**					
±0.50 D (%)	66.67	62.96	70.37	66.67	70.37
±1.00 D (%)	74.10	70.40	77.80	74.10	74.10
MAE ^1^	0.465	0.534	**0.493**	0.491	0.496
MedAE ^2^	0.230	0.290	0.275	0.240	0.300

^1^ Mean absolute prediction error; ^2^ median absolute prediction error; ^3^ Barrett True-K no history; ^4^ EVO 2.0 Post-Hyperopic LASIK/PRK.

## Data Availability

The data presented in this study are available on request from the corresponding author.
